# *IGHV*1-69 B Cell Chronic Lymphocytic Leukemia Antibodies Cross-React with HIV-1 and Hepatitis C Virus Antigens as Well as Intestinal Commensal Bacteria

**DOI:** 10.1371/journal.pone.0090725

**Published:** 2014-03-10

**Authors:** Kwan-Ki Hwang, Ashley M. Trama, Daniel M. Kozink, Xi Chen, Kevin Wiehe, Abby J. Cooper, Shi-Mao Xia, Minyue Wang, Dawn J. Marshall, John Whitesides, Munir Alam, Georgia D. Tomaras, Steven L. Allen, Kanti R. Rai, Jane McKeating, Rosa Catera, Xiao-Jie Yan, Charles C. Chu, Garnett Kelsoe, Hua-Xin Liao, Nicholas Chiorazzi, Barton F. Haynes

**Affiliations:** 1 Duke Human Vaccine Institute, Duke University Medical Center, Durham, North Carolina, United States of America; 2 School of Immunity and Infection, Institute of Biomedical Research, University of Birmingham, Birmingham, United Kingdom; 3 The Karches Center for Chronic Lymphocytic Leukemia Research, The Feinstein Institute for Medical Research, Manhasset, New York, United States of America; 4 Department of Immunology, Duke University School of Medicine, Durham, North Carolina, United States of America; University of Massachusetts Medical Center, United States of America

## Abstract

B-cell chronic lymphocytic leukemia (B-CLL) patients expressing unmutated immunoglobulin heavy variable regions (*IGHV*s) use the *IGHV*1-69 B cell receptor (BCR) in 25% of cases. Since HIV-1 envelope gp41 antibodies also frequently use *IGHV*1-69 gene segments, we hypothesized that *IGHV*1-69 B-CLL precursors may contribute to the gp41 B cell response during HIV-1 infection. To test this hypothesis, we rescued 5 *IGHV*1-69 unmutated antibodies as heterohybridoma IgM paraproteins and as recombinant IgG_1_ antibodies from B-CLL patients, determined their antigenic specificities and analyzed BCR sequences. *IGHV*1-69 B-CLL antibodies were enriched for reactivity with HIV-1 envelope gp41, influenza, hepatitis C virus E2 protein and intestinal commensal bacteria. These *IGHV*1-69 B-CLL antibodies preferentially used *IGHD*3 and *IGHJ*6 gene segments and had long heavy chain complementary determining region 3s (HCDR3s) (≥21 aa). *IGHV*1-69 B-CLL BCRs exhibited a phenylalanine at position 54 (F_54_) of the HCDR2 as do rare HIV-1 gp41 and influenza hemagglutinin stem neutralizing antibodies, while *IGHV*1-69 gp41 antibodies induced by HIV-1 infection predominantly used leucine (L_54_) allelic variants. These results demonstrate that the B-CLL cell population is an expansion of members of the innate polyreactive B cell repertoire with reactivity to a number of infectious agent antigens including intestinal commensal bacteria. The B-CLL *IGHV*1-69 B cell usage of F_54_ allelic variants strongly suggests that *IGHV*1-69 B-CLL gp41 antibodies derive from a restricted B cell pool that also produces rare HIV-1 gp41 and influenza hemagglutinin stem antibodies.

## Introduction

The initial B cell responses to HIV-1 envelope (Env) gp41 are non-neutralizing [1] and are polyreactive with human intestinal commensal bacterial antigens [2]. Env gp41 antibodies that arise following HIV-1 transmission do not select virus escape mutants and therefore exert no anti-viral immune pressure [1]. We have recently demonstrated that gp41-reactive B cells can be isolated prior to infection in HIV-1-uninfected humans and that HIV-1 activates preexisting B cells that are cross-reactive with gp41 and non-HIV-1 antigens including microbial antigens [2]. However, the pool of B cells from which the initial HIV-1 Env B cell response is derived is not known.

B chronic lymphocytic leukemia (B-CLL) is a clonal expansion of CD5^+^ B lymphocytes frequently associated with unmutated B cell receptors (BCRs) [3]. B-CLL cells with unmutated immunoglobulin heavy variable regions (*IGHVs*) (unmutated CLL, U-CLL) show a preferential usage of *IGHV*1-69 gene segment (∼25%) and frequently have BCRs that are polyreactive and autoreactive despite dramatic structural restrictions [4]–[10]. The cellular origin of B-CLL cells has been an area of considerable debate. For example, it has been proposed that B-CLL cells derive from human B-1-like cells, marginal zone (MZ) innate B cells, or transitional B cells, based on cell surface phenotype and molecular and functional characteristics [11]. In this regard, recent studies identified a human equivalent of murine B-1 cells (CD20^+^, CD27^+^, CD43^+^, CD70^−^) [12] and circulating CD5^+^ human B cells [13] as the precursors of CLL B cells. It has also been proposed that B-CLL cells with BCR stereotypy could derive from B-1-like progenitor cells adapted to particular antigenic challenges while B-CLL cells with heterogeneous BCRs could derive from conventional B cells [14]. In addition, anti-viral innate antibodies have been reported to be derived from B-1/MZ B cells [15]–[17].

The *IGHV*1-69 BCR allelic variants expressed in B-CLL cells predominantly use a phenylalanine at position 54 (F_54_) of the heavy chain complementarity determining region 2 (HCDR2) [18], [19] and the estimated global frequency of F_54_ is 60% [20]. Interestingly, influenza antibodies (e.g. CR6261, F10, FE53, and 1009-3B05) [21]–[24] and HIV-1 antibodies (e.g. D5, HK20, and Fab 8066) [25]–[27] that bind to the hemagglutinin stem or HIV-1 gp41 regions, respectively, have been reported to use F_54_ *IGHV*1-69 allelic variants, and in the case of influenza antibodies, such F_54_ antibodies are broadly neutralizing [21]–[24]. The F_54_ *IGHV*1-69 allelic variants showed a common binding mode to gp41 heptad repeat (HR)-1 coiled-coil hydrophobic pocket [25]–[27]. Thus, in this study we hypothesized that *IGHV*1-69 B-CLL precursors may contribute to the gp41 B cell response during HIV-1 infection. We identified B-CLL *IGHV*1-69 antibodies cross-reacting with viral and commensal bacterial antigens, evaluated their association with clinical outcome of B-CLL patients, and compared the immunoglobulin gene characteristics with those of HIV-1 gp41 antibodies isolated from HIV-1 infection.

## Methods

### Ethics statement

Specimens collected from B-CLL patients were obtained under an Institutional Review Board (IRB) approved protocol at The Feinstein Institute for Medical Research, North Shore – LIJ Health System, Manhasset, NY and were part of The Feinstein's Center for CLL Research Biorepository. The research was conducted according to the principles expressed in the Declaration of Helsinki. Written informed consent was obtained from all subjects.

De-identified PBMCs from B-CLL patients not collected for the purpose of this research were obtained from The Feinstein's Center for CLL Research Biorepository under a Duke IRB exemption and fully executed material transfer agreement. Normal donors were collected under a Duke IRB approved protocol. The research was conducted according to the principles expressed in the Declaration of Helsinki. Written informed consent was obtained from all subjects.

The Duke University Health System Institutional Review Board for Clinical Investigations (DUHS IRB), is duly constituted, fulfilling all requirements for diversity, and has written procedures for initial and continuing review of human research protocols. The DUHS IRB complies with all U.S. regulatory requirements related to the protection of human research participants. Specifically, the DUHS IRB complies with 45CFR46, 21CFR50, 21CFR56, 21CFR312, 21CFR812, and 45CFR164.508–514. In addition, the DUHS IRB complies with the Guidelines of the International Conference on Harmonization to the extent required by the U.S. Food and Drug Administration.

### Cell culture

Epstein-Barr virus (EBV)-stimulation of patient peripheral blood mononuclear cells (PBMCs) and generation of B-CLL hetero-hybridoma cell lines have been described previously [28]. We stimulated PBMCs from 58 B-CLL patients (33 *IGHV*1-69 and 25 *IGHV*2/*IGHV*3) with EBV in the presence of a Toll-like receptor 9 agonist ODN2006 (12.5 µg/ml; Invivogen) and cyclosporin A (0.5 µg/ml), and cultured the cells in the presence of feeder cells, J774A.1 (50,000 cells per well; American Type Culture Collection, TIB-67) that had been exposed to γ-irradiation (40 Gy) from a Shepherd irradiator. Three weeks after stimulation, culture supernatant was collected from each well, and levels of total IgM were measured using a previously described method [28]. We obtained 39 patient cultures (22 *IGHV*1-69 and 17 *IGHV*2/*IGHV*3) that produced similar levels of IgM. Of the 22 *IGHV*1-69 samples, 21 were U-CLL and 1 mutated CLL (M-CLL) while of the 17 *IGHV*2/*IGHV*3 samples, 9 were U-CLL and 8 M-CLL (**Table S1**). As negative controls, EBV-stimulated B cell cultures from PBMCs of 20 normal subjects were studied.

### Binding assays for screening and characterization of monoclonal antibody (mAb) specificity

Culture supernatants or purified mAbs were assayed for reactivity to a panel of test antigens by ELISA [29]. HIV-1 antigens included AT-2-inactivated HIV-1 virions ADA (clade B) [30], HIV-1 group M consensus envelope, ConS gp140 [31], deglycosylated JRFL gp140 [32], HIV-1 MN recombinant gp41 (Immunodiagnostics), a gp41 HR-1 region peptide, DP107 (NNLLRAIEAQQHLLQLTVWGIKQLQARILAVERYLKDQ), an Env clade B HR-2 region peptide, MPER656 (NEQELLELDKWASLWNWFNITNWLW), an Env clade C HR-2 region peptide, MPR.03 (KKKNEQELLELDKWASLWNWFDITNWLWYIRKKK), and an Env clade B immunodominant region peptide, SP400 (RVLAVERYLRDQQLLGIWGCSGKLICTTAVPWNASWSNKSLNKI). Use of *IGHV*1-69 gene segment is high among neutralizing antibodies against hepatitis C virus (HCV) [33] and influenza stem antibodies [22], [34]. Thus, we also used non-HIV-1 antigens including recombinant HCV E2 protein (subtype 1a; Immunodiagnostics), trivalent influenza vaccine (Fluzone 2008; Sanofi Pasteau), and two hemagglutinin glycoproteins, A/Solomon Islands (H1N1) and B/Florida (Sanofi Pasteau).

Briefly, ELISA plates (Costar, Cambridge, MA) were coated with 1–5 µg/ml of test antigens in 0.1 M sodium bicarbonate buffer. After incubating overnight at 4°C, plates were blocked with PBS containing 15% goat serum, 4% whey protein, 0.5% Tween-20, and 0.05% NaN_3_. Then test supernatants or mAbs diluted in the blocking buffer were distributed to wells and incubated for 2 hours at room temperature. After washing with PBS-0.5% Tween-20, bound human IgM or IgG was detected with horseradish peroxidase-conjugated goat anti-human IgM or IgG (μ-chain or γ-chain specific; Jackson ImmunoResearch Laboratories, West Grove, PA) and peroxidase substrate tetramethylbenzidine (Kirkegaard and Perry Laboratories, Gaithersburg, MD) using a SpectraMax Plus384 plate reader (Molecular Devices, Sunnyvale, CA). The detection limit of IgM in each well was 60 ng/ml; negative wells with undetectable levels of IgM were assigned 10 ng/ml to permit logarithmic transformation of the data.

Reactivity of mAbs to aerobic and anaerobic bacteria whole cell lysates was tested by binding antibody multiplex (Luminex) assays as previously described [1], [2]. Bacterial whole cell lysates were prepared using previously described methods [2], [35]. In addition, surface plasmon resonance analysis of mAb reactivity to MN gp41 and HCV E2 proteins was performed on a BIAcore 3000 (BIAcore Inc.) using the methods as previously described [2].

### Expression of recombinant IgG_1_ mAbs

Live EBV-stimulated B cells from selected wells were sorted as single cells using a BD FACS Aria (BD Biosciences, San Jose, CA), and the isolated VH and VL gene pairs were assembled by PCR into the linear full-length immunoglobulin heavy- and light-chain gene expression cassettes for production of recombinant IgG_1_ mAbs by transfection in the human embryonic kidney cell line, 293F (American Type Culture Collection) using the methods as previously described [29].

## Results

### Reactivity of *IGHV*1-69 and non-*IGHV*1-69 B-CLL cells with HIV-1 and other antigens

To compare binding activities of B-CLL IgMs expressing *IGHV1-69* vs. *IGHV*2/*IGHV*3 gene families, we stimulated PBMCs from 58 B-CLL patients (33 *IGHV*1-69 and 25 *IGHV*2/*IGHV*3) with EBV as described previously [28]. Three weeks after stimulation, we obtained 39 patient cultures (22 *IGHV*1-69 and 17 *IGHV*2/*IGHV*3) that produced similar levels of IgM. The mean IgM levels were 2.1 µg/ml (range, 0.13–10.1 µg/ml) and 2.1 µg/ml (range, 0.16–8.0 µg/ml) for *IGHV*1-69 and *IGHV*2/*IGHV*3 groups, respectively. As negative controls, EBV-stimulated B cell cultures from PBMCs of 20 normal subjects were studied. The mean IgM level of the control group was 6.7 µg/ml (range, 1.0–13.3 µg/ml).

The *IGHV*1-69 B-CLL PBMC culture supernatants were highly enriched for HIV-1 Env reactivity. Of 22 *IGHV*1-69 B-CLL patient samples, 6 (27.3%) reacted with HIV-1 Envs; 3 (13.6%) reacted with ADA aldrithol-2 (AT-2)-inactivated virions, 1 (4.5%) with deglycosylated JRFL gp140, and 4 (18.2%) with one or more gp41 epitope peptides. In contrast, none of 17 *IGHV*2/*IGHV*3 B-CLL (p = 0.027; Fisher's exact test), or 20 normal control samples (p = 0.022; Fisher's exact test) reacted with HIV-1 Env antigens (**Figure S1** and **Figure S2**). Of 22 *IGHV*1-69 B-CLL patient samples, 7 (31.8%) reacted with HCV E2 recombinant protein while only 2 of 17 (11.8%) *IGHV*2/*IGHV*3 (p = 0.25; Fisher's exact test) and none of 20 normal control samples (p = 0.009; Fisher's exact test) reacted with HCV E2. Similarly, 6 of 22 (27.3%) *IGHV*1-69 B-CLL samples reacted with trivalent influenza vaccine while 2 of 17 (11.8%) *IGHV*2/*IGHV*3 (p = 0.43; Fisher's exact test) and 3 of 20 (15.0%) normal control samples (p = 0.46; Fisher's exact test) were trivalent influenza vaccine-reactive (**Table S2**).

### Production and characterization of B-CLL mAbs

**Figure 1:**
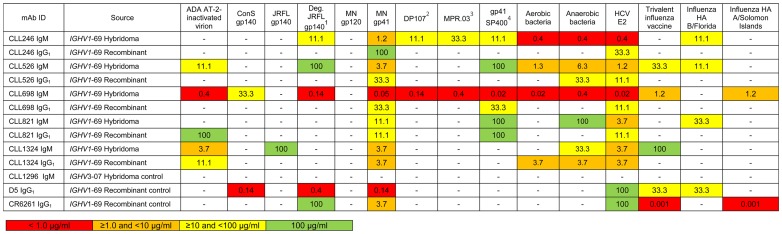
Reactivity of IgM paraproteins produced by B-CLL hetero-hybridomas and the corresponding recombinant IgG_1_ mAbs. Values are representative endpoint concentrations (in μg/ml) from at least two separate experiments. ^1^Deglycosylated JRFL gp140; ^2^HIV-1 gp41 HR-1 region peptide, DP107 sequence (NNLLRAIEAQQHLLQLTVWGIKQLQARILAVERYLKDQ); ^3^HIV-1 envelope clade C gp41 HR-2 region peptide, MPR.03 sequence (KKKNEQELLELDKWASLWNWFDITNWLWYIRKKK); ^4^HIV-1 BAL gp41 immunodominant region, SP400 sequence (RVLAVERYLRDQQLLGIWGCSGKLICTTAVPWNASWSNKSLNKI); Aerobic/anaerobic intestinal commensal bacterial whole-cell lysates. A HIV-1 gp41 antibody, D5 [26] and an influenza HA antibody, CR6261 [22] were included as positive controls. “-” denotes no binding.

To test gp41 reactivity of B-CLL purified IgMs, we rescued 5 *IGHV*1-69 B-CLL mAbs as hetero-hybridoma IgM paraproteins; CLL246, CLL526, CLL698, CLL821, and CLL1324 (all U-CLL) as described previously [28]. A HIV-1-negative mAb (CLL1296; *IGHV*3-07, 7.6% difference from the germline) was used as a negative control [28]. All 5 *IGHV*1-69 unmutated IgMs reacted with HIV-1 clade B MN gp41 recombinant protein while none reacted with HIV-1 MN gp120 Env glycoprotein (**Figure 1**). We have previously demonstrated that deglycosylation of native Env exposes gp41 epitopes [32]. CLL246, CLL526, and CLL698 IgMs reacted with deglycosylated HIV-1 JRFL gp140 but not glycosylated gp140, indicating that Env glycosylation interfered with their reactivity. The CLL246 and CLL698 IgMs also reacted with each of 3 gp41 linear epitope peptides, DP107, MPR.03, and SP400, indicating the polyreactive nature of the pentameric IgMs (**Figure 1**).

**Figure 2:**
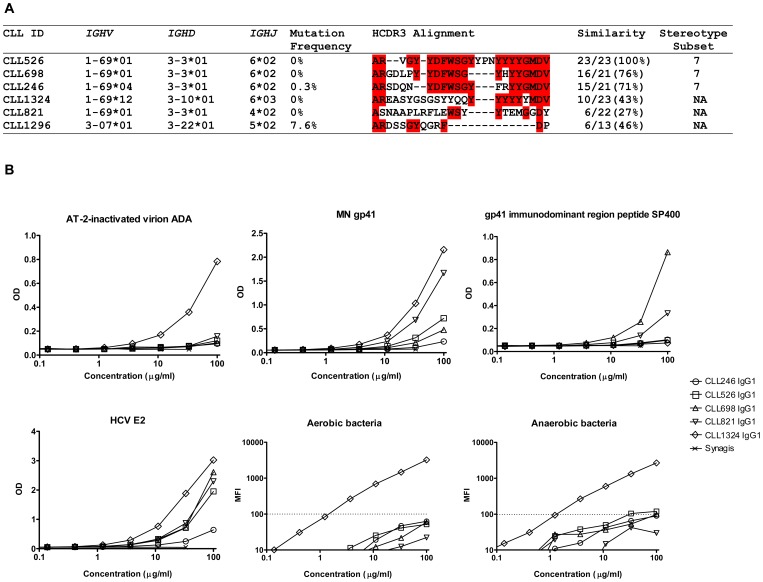
Sequence and binding characteristics of HIV-1 Env gp41-reactive *IGHV*1-69 B-CLL mAbs. (A) The gp41-reactive *IGHV*1-69 B-CLL mAbs were unmutated and preferentially used *IGHD*3-3 and *IGHJ*6 gene segments. The aa sequences of the HCDR3 regions of 5 gp41-reactive *IGHV*1-69 B-CLL IgMs were aligned to that of CLL526 IgM. Each sequence was aligned independently to CLL526 (pairwise alignment) using ClustalW and final adjustment was made manually. Gaps are indicated as dashes. The aa conserved between the sequences of CLL526 and the other IgMs are highlighted in red. The number of aa shared with CLL526 over the total aa is reported on the right for each IgM. The CLL1296 IgM was used as a negative control. (B) Binding characteristics of the B-CLL mAbs expressed as recombinant IgG_1_ with HIV-1, HCV, and intestinal commensal bacterial antigens. Serial dilutions ranging from 100 µg/ml to 0.004 µg/ml of each IgG were tested in ELISA for binding to ADA AT-2-inactivated virion, MN gp41, HIV-1 BAL gp41 immunodominant region peptide SP400 (RVLAVERYLRDQQLLGIWGCSGKLICTTAVPWNASWSNKSLNKI), and HCV E2, or in Luminex assay for aerobic and anaerobic intestinal commensal bacterial whole-cell lysates. Data are expressed in OD for ELISA or mean fluorescence intensity (MFI) for Luminex assay. The dotted lines indicate the cut-off value ≥100 MFI used to denote positivity. Data are representative of at least two separate experiments.

The 5 gp41-reactive B-CLLs expressed *IGHV* genes that were completely or nearly unmutated (**Figure 2A**). CLL246, CLL526, and CLL698 belong to the subset 7 of stereotypic rearrangements of BCRs described in B-CLL [14], and all but CLL821 used D region reading frame 2 that are enriched for hydrophobic amino acids (**Table S1**) [36]. Four of 5 gp41-reactive B-CLLs used *IGHD*3-3 gene segment and 4 of 5 used *IGHJ*6 gene segment. Further, all expressed long HCDR3s (21-23 aa) (**Figure 2A**). Of note, all but CLL246 used the F_54_ *IGHV*1-69 allelic variants.

We next expressed the 5 B-CLL mAb V_H_DJ_H_ and V_L_J_L_ genes as full-length IgG_1_ recombinant mAbs [29]. All 5 B-CLL recombinant IgGs bound to MN gp41 (**Figure 2B**). Of these, CLL698 and CLL821 IgGs bound to the immunodominant region of HIV-1 clade B BAL gp41 (RVLAVERYLRDQQLLGIWGCSGKLICTTAVPWNASWSNKSLNKI) (**Figure 2B**). However, CLL246 and CLL698 IgGs did not bind to any other linear peptides tested including DP107, MPR.03, MEPR656, and overlapping 15-mer MN gp41 linear peptides (data not shown). These results indicated that multivalent IgM antibodies with high avidity interactions could enhance low affinity interactions between the unmutated IgG antibodies and the linear peptides tested.

**Figure 3:**
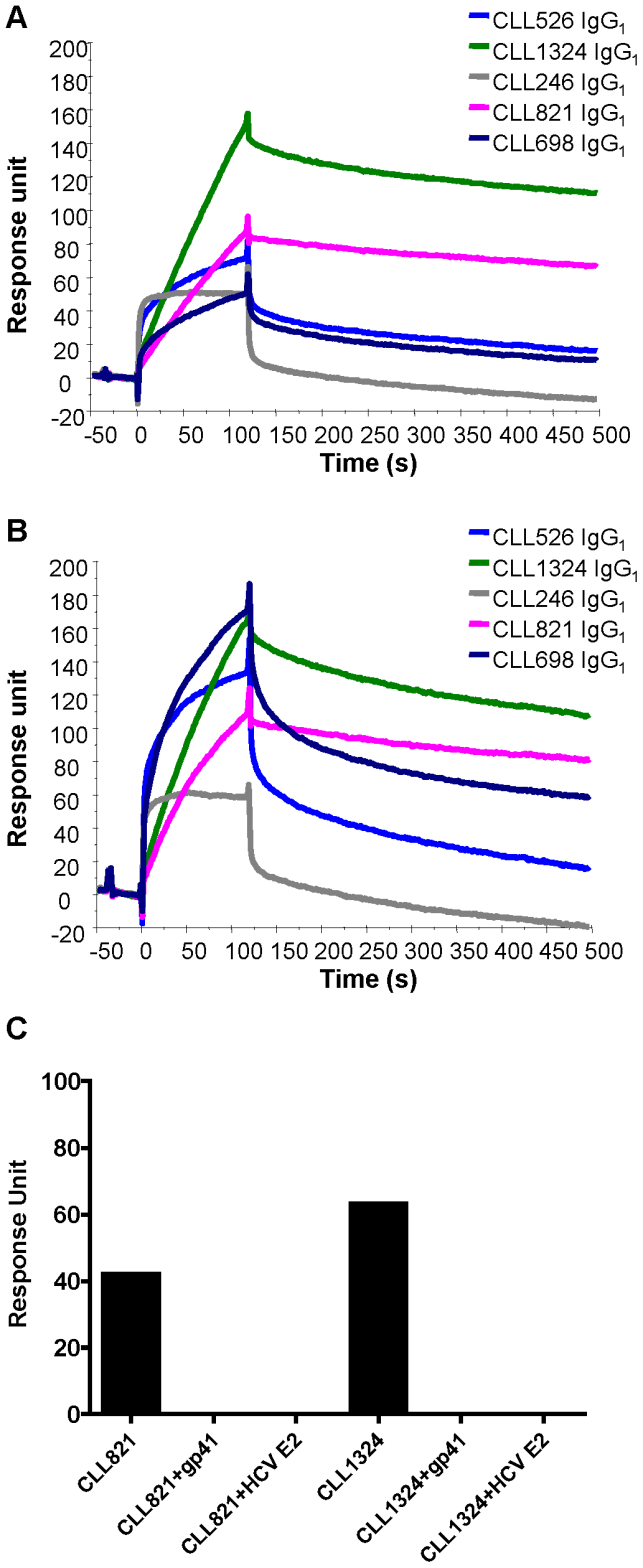
Binding of recombinant B-CLL IgG1 mAbs to MN gp41 and HCV E2 proteins in surface plasmon resonance binding assays. MN gp41 (A) or HCV E2 (B) protein was captured on a sensor chip surface and test mAbs were injected over each of the test antigens. Test mAbs preincubated with either MN gp41 or HCV E2 proteins were injected over MN gp41 immobilized on a sensor chip surface (C). Data are expressed in response unit of binding to MN gp41.

Gp41 antibodies that arise in HIV-1 infection frequently cross-react with intestinal commensal bacterial antigens and indeed have been postulated to derive from pre-transmission environmental antigen-reactive antibodies from memory B cells [2]. Therefore, we tested reactivity of B-CLL mAbs with aerobic and anaerobic intestinal commensal bacterial whole-cell lysates using binding antibody multiplex assays [2]. We found all 5 *IGHV*1-69 unmutated IgMs reacted with aerobic and/or anaerobic intestinal commensal bacterial whole-cell lysates (**Figure 1**). The recombinant IgGs of CLL526 and CLL1324 also reacted with aerobic and/or anaerobic intestinal commensal bacterial whole-cell lysates (**Figure 2B**). Similarly, all 5 *IGHV*1-69 unmutated IgMs and their recombinant IgGs also reacted with HCV E2 protein (**Figure 1** and **Figure 2B**). Two mAbs were chosen for cross-competition studies with HCV E2; recombinant E2 competitively inhibited the binding of CLL821 and CLL1324 IgGs to gp41 (**Figure 3**).

It has been proposed that B-CLL cells derive from autoreactive B cell precursors [6], [37]. In this regard, 2 of 5 recombinant IgG mAbs (CLL698 and CLL1324) bound to double-stranded DNA but not to the other test autoantigens including SSA, SSB, Sm, RNP, Scl-70, Jo-1, centromere B, and histone (data not shown). In our indirect immunofluorescence staining assay, however, none of the IgM paraproteins or the recombinant IgG mAbs reacted with HEp-2 epithelial cells, and none showed rheumatoid factor activity (data not shown). In functional assays, none of the IgM or IgG B-CLL mAbs neutralized HIV-1 strains, SF162 (clade B), BG1168 (clade B), or MN (clade B) (**Table S3**) [2]. Similarly, none of the IgM mAbs inhibited syncytium formation by HIV-1 ADA (clade B) and MN nor captured HIV-1 virions, SF162 or BG1168 (**Table S4** and **Table S5**). Moreover, none of the IgMs neutralized a HCV subtype 1a strain, HCVpp-H77 (**Table S3**) [38].

### Comparison of gp41-reactive *IGHV*1-69 B-CLL mAbs with *IGHV*1-69 gp41 antibodies from HIV-infected patients

It has been previously reported that the *IGHV*1-69 B-CLL BCRs predominantly use *IGHV*1-69 allotypic variants with F_54_ [18], [19] while the estimated global frequency of F at this position is 60% [20]. We studied a series of gp41 antibodies isolated from acute or chronic HIV-1 infected subjects and found that of the 116 gp41 antibodies, 40.5% (47/116) were *IGHV*1-69 compared to 5.4% (32/595) of non-HIV-1-reactive antibodies isolated either from HIV patient plasma cells or from memory B cells (**Table 1**). While 86.8% (125/144) of *IGHV*1-69 B-CLL BCRs in our laboratory's database of B-CLL sequences from 1089 patients were F_54_ allelic variants, 87.2% (41/47) of the *IGHV*1-69 gp41 antibodies from HIV-infected subjects were L_54_ allelic variants (**Table 1**). Thus, while both B-CLL antibodies and HIV-1 gp41 antibodies are enriched in *IGHV*1-69 antibodies [39], [40], B-CLL BCRs predominantly use F_54_, while HIV-1 infection recruits predominantly L_54_ *IGHV*1-69 allelic variant B cells to respond to HIV-1.

**Table 1:** *IGHV1*-69 allelic variants used by gp41 mAbs upon HIV-1 infection.

Cohort	mAb specificity	No. of subjects (N)	Total mAbs	*IGHV1*-69	F_54_	L_54_	Other
HIV-1 infected	gp41	15	116	47 (40.5%)	1 (2.1%)	41 (87.2%)	5 (10.6%)
HIV-1 infected	Non-HIV	17	595	32 (5.4%)	19 (59.4%)	6 (18.8%)^2^	7 (21.9%)
B-CLL^1^	nd	1089	1139	144 (12.6%)	125 (86.8%)	17 (11.8%)^2^	2 (1.4%)

1 All sequences are available in the IMGT and GenBank databases. ^2^p<0.0001 versus gp41-reactive *IGHV1*-69 antibodies isolated from HIV-1-infected subjects. Nd, not determined.

**Figure 4:**
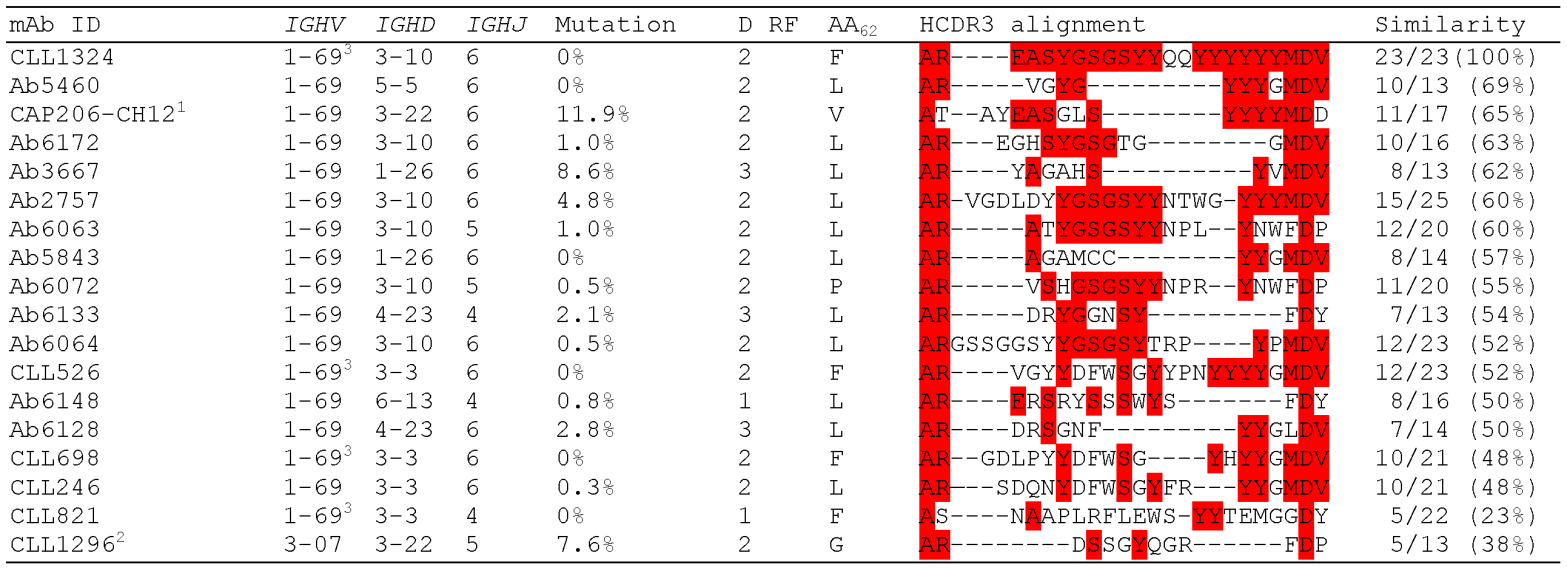
HCDR3 alignment of CLL1324 to gp41-reactive *IGHV*1-69 antibodies isolated from HIV-1-infected patients. The aa sequences of the HCDR3 regions of gp41-reactive *IGHV*1-69 antibodies isolated from HIV-1-infected patients were aligned to that of CLL1324. Each sequence was aligned independently to CLL1324 (pairwise alignment) using ClustalW and final adjustment was made manually. Gaps are indicated as dashes. The aa conserved between the sequences of CLL1324 and the other antibodies are highlighted in red. The number of aa shared with CLL1324 over the total aa is reported on the right for each antibody. Only the gp41 antibody sequences with HCDR3 % similarity ≥50% are reported. The CLL1296 IgM was used as a negative control. ^1^Previously published sequence [51]; ^2^CLL1296, HIV-1-negative control mAb; ^3^ *IGHV*1-69 antibodies with an F_54_ allelic variant. D RF, D gene reading frame; AA_54_, aa in position 54.

The HCDR3 sequences are the principal determinants of antibody-binding specificity in most antibodies [41]. Thus, we compared HCDR3 sequences of the 5 gp41-reactive *IGHV*1-69 B-CLLs with those of 47 gp41-reactive *IGHV*1-69 antibodies isolated from HIV-1-infected patients. The analysis revealed similar HCDR3 sequences due to common usages of *IGHJ*6 and *IGHD*3 gene segments that were preferentially used by gp41-reactive B-CLL mAbs. For example, the long HCDR3 sequences of mAbs Ab2757 (25 aa) and Ab6064 (23 aa) were remarkably similar (60% and 52% aa identity, respectively) to that of CLL1324 (**Figure 4**). However, *IGHJ*4 was the most frequently used gene segment (32%) in the HIV-1 infection-derived *IGHV*1-69 gp41 antibodies in contrast to the infrequent use of *IGHJ*4 by *IGHV*1-69 B-CLL (∼4%) [18]. In addition, *IGHD*3-3, the most frequent D gene segment used by the gp41-reactive B-CLL mAbs was found in only 4% (2/47) of the HIV-1 infection-derived *IGHV*1-69 gp41 antibodies. The mean HCDR3 length of the HIV-1 infection-derived *IGHV*1-69 gp41 antibodies was significantly shorter than that of the gp41-reactive *IGHV*1-69 B-CLL antibodies (16.1 aa vs. 22 aa; Mann-Whitney test, p = 0.0041). Moreover, the sequence pattern cluster analysis of HCDR3s indicated that none of the HIV-1 infection-derived *IGHV*1-69 gp41 antibodies belonged to the known major B-CLL stereotype subsets [14]. These results indicate that the gp41-reactive *IGHV*1-69 CLL B cells have molecular features distinct from those found in most *IGHV*1-69 gp41 B cells during HIV-1 infection.

### Virus binding activity of B-CLL and clinical outcomes

**Figure 5:**
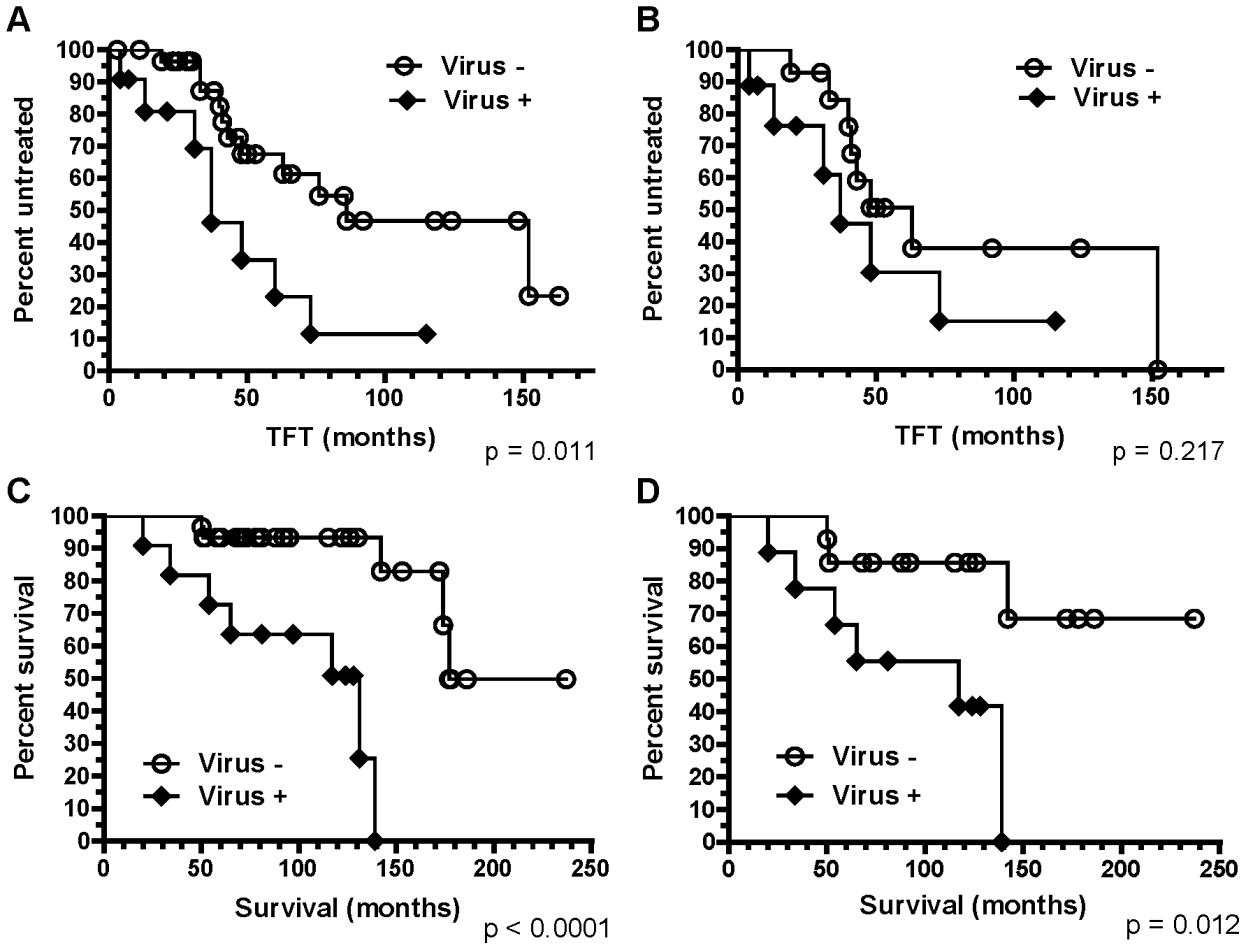
B-CLL cases with anti-viral reactivity correlate with poor clinical outcomes. The Kaplan-Meier plots are shown for the time to first treatment (TFT, in months) in all samples (A) and *IGHV*1-69 samples (B). The p values for Mantel-Cox test in groups A and B are 0.011 and 0.217, respectively. The Kaplan-Meier plots are shown for overall patient survival (in months) in all samples (C) and *IGHV*1-69 samples (D). The p values for Mantel-Cox test in groups C and D are <0.0001 and 0.012, respectively. Virus+ group represents B-CLL samples with ≥10 wells out of 20 wells tested showing a specific anti-viral reactivity (**Figure S1**). The results for virus-binding activity of 2 B-CLL samples (CLL821 and CLL1296) were obtained from the purified IgM paraproteins (**Figure 1**).

When we divided the B-CLL samples based on their binding activity to the test viral antigen preparations (**Figure S1**), we found that virus antigen-binding reactivity of B-CLL cultures correlated with B-CLL clinical course. The Kaplan-Meier plots of the analyses revealed that B-CLL cases with anti-viral reactivity correlated with poor clinical outcomes measured as time to first treatment (TFT) and overall survival of the patients (**Figure 5**). The median TFTs for virus-binding and non-virus binding groups were 37 mo and 86 mo, respectively (p = 0.011, Mantel-Cox test), and the median overall survival for virus binding and non-virus binding groups were 131 mo and 177 mo, respectively (p<0.0001, Mantel-Cox test). This was especially impressive when restricting the analysis to *IGHV*1-69 samples (**Figure 5B** and **Figure 5D**). The median overall survival for virus binding and non-virus binding groups were 117 mo and indefinite, respectively (p = 0.012, Mantel-Cox test). Of note, all but one (CLL1011) *IGHV*1-69 samples were U-CLL and would therefore be expected to have poor clinical outcome [3]. However, the U-CLL *IGHV*1-69 samples could be segregated by virus binding activity, with the non-binders to viral antigens having good clinical outcome. These findings suggested that certain BCRs with innate anti-viral reactivity may be important factors in determining the outcome of the B-CLL clinical course.

## Discussion

In this paper, we have demonstrated that one third of *IGHV*1-69 B-CLL BCRs are polyreactive for infectious agent or commensal bacterial antigens (**Figure S1** and **Figure 1**). B-CLL IgM reactivity with infectious agent antigens was significantly correlated with poor clinical outcomes (**Figure 5**). Moreover, there was a striking difference in *IGHV*1-69 allelic use by B-CLL versus HIV-1 *IGHV*1-69 antibodies. While *IGHV*1-69 B-CLL BCRs predominantly used F_54_ allelic variants, *IGHV*1-69 HIV-1 Env gp41 antibodies from HIV-1 infected patients predominantly used L_54_ (**Table 1**).

Liao et al. [2] have demonstrated that the initial blood plasma cell response in acute HIV-1 infection to gp41 is highly mutated and comprised of polyreactive gp41 antibodies that cross-react with intestinal commensal bacterial antigens. This work led to the hypothesis that the initial gp41 response to HIV-1 may be in part derived from commensal bacteria-activated memory B cells with BCRs that cross-react with Env gp41 and not from naïve B cells [2]. Thus, HIV-1 Env in the context of HIV-1 infection induces a dominant Env gp41 antibody response that is polyreactive with host and intestinal commensal bacterial antigens [2]. The observation that *IGHV*1-69 B-CLL BCRs are similarly polyreactive and cross-react with intestinal commensal bacteria (**Figure 1**) raises the hypothesis that the B-CLL cell population is an expansion of members of the innate polyreactive B cell repertoire with reactivity to a number of infectious agent antigens including intestinal commensal bacteria. Hence, our results suggested that the initial response to gp41 in HIV-1 may derive from the same pool of B cells as B-CLL. However, it is striking that B-CLL B cells predominantly utilize F_54_ *IGHV*1-69 allelic variants while HIV-1 Env gp41 B cell BCRs from HIV-1 infection utilize L_54_ allelic variants (**Table 1**). Therefore, the B-CLL *IGHV*1-69 B cell usage of F_54_ allelic variants demonstrate that the initial response to gp41 in HIV-1 may not derive from the same pool of B cells as B-CLL. In fact, the B-CLL *IGHV*1-69 B cells may drive from an F_54_ allelic variant B cell pool that produces rare gp41 and hemagglutinin stem antibodies. It has been demonstrated that the F_54_ *IGHV*1-69 allelic variant B cells arise during early human fetal liver development [42]. They were found in a high proportion of B cells in the primary follicles of fetal spleen [43] and in the mantle zones of adult tonsil [44]. Thus, B-CLL B cells may derive from this mantle zone pool of polyreactive B cell precursors [18], [45], [46].

The 5 gp41-reactive unmutated B-CLL mAb clones had similar HCDR3 sequences due to common *IGHV-D-J* rearrangements, and as well, had long HCDR3s (21–23 aa) (**Figure 2A**). Three clones (CLL246, CLL526, and CLL698) belong to subset 7 according to the major stereotyped BCR subset numbering based on a sequence pattern cluster analysis of B-CLL HCDR3s (**Figure 2A**) [14]. Unmutated B-CLL B cells with stereotypy give rise to the hypothesis that they are derived from a subset of B cells selected for ability to bind to bacterial and viral antigens, characteristics of B-1, transitional and MZ B cells [11]. It has been proposed that a small population of CD20^+^CD27^+^CD43^+^CD70^-^ cells present in human umbilical cord and adult peripheral blood represent a B cell subset analogous to the murine B-1 subset [12], and human transitional and MZ B cells share traits that are similar to murine B-1 B cells, and collectively produce pre-formed antibodies to pathogens [47].

For both HIV-1 and HCV, we found no neutralizing antibodies among any of the B-CLL gp41 or HCV E2-reactive antibodies. Similarly, acute HIV-1 infection gp41 antibodies are non-neutralizing [1], [2]. In contrast, the influenza-reactive non-mutated *IGHV*1-69 antibodies F10 and CR6260 neutralized a broad spectrum of influenza strains [21], [24]. If IgM antibodies can coat infectious agent virions, they may impede virus migration across mucosal surfaces [48], [49]. However, virus capture assays showed that none of gp41-reactive B-CLL mAbs captured test HIV-1 virions. Moreover, acute HIV-1 infection gp41 antibodies do not exert immune pressure via selecting escape mutants [1].

Finally, several studies have shown that unmutated B-CLL B cells, similar to natural or innate IgM antibodies, frequently express polyreactive antibodies that bind to autoantigens associated with apoptosis and oxidation as well as to components of the outer membrane of bacteria [37], [50]. Of note, it has been demonstrated that human B-1-like cells (CD20^+^CD27^+^CD43^+^CD70^−^) displayed a skewed BCR repertoire as indicated by preferential expression of anti-phosphorylcholine and anti-DNA specificities [12]. Our findings that unmutated B-CLL cell gp41 reactivity is selective for the F_54_ *IGHV*1-69 gene segment and has characteristics of B-1-like, transitional and MZ B cell derived antibodies strongly suggest that B-CLL *IGHV*1-69 gp41 antibodies derive from a restricted B cell pool that also produces rare HIV-1 gp41 and influenza hemagglutinin stem antibodies.

## Supporting Information

Figure S1 **Binding characteristics of B-CLL B cell cultures.** To compare binding activities of B-CLL IgMs expressing *IGHV1*-69 vs. *IGHV*2/*IGHV*3 gene families, we stimulated PBMCs from B-CLL patients with EBV using the methods as previously described [28], and the cells were plated at 5,000 cells per well in total of 20 wells per patient sample. To profile binding characteristics of IgMs, we screened the culture supernatants in ELISA. HIV-1 antigens included aldrithol-2 (AT-2)-inactivated HIV-1 virions ADA (Clade B); HIV-1 group M consensus Env, ConS gp140; and deglycosylated JRFL gp140. HIV-1 Env gp41 linear epitope peptides included HR-1 region peptide, DP107 (NNLLRAIEAQQHLLQLTVWGIKQLQARILAVERYLKDQ); Env clade B HR-2 region peptide, MPER656 (NEQELLELDKWASLWNWFNITNWLW); and Env clade C HR-2 region peptide, MPR.03 (KKKNEQELLELDKWASLWNWFDITNWLWYIRKKK). As an initial approach to ensure reactivity of IgMs were of B-CLL origin, rather than IgMs from contaminating B cells, we defined positive samples as they produced 10 or more wells (≥50%) reactive with each test antigen. Of 440 *IGHV1*-69 B-CLL cultures from 22 patients, 67 wells reacted with DP107, 20 reacted with the MPER656, and 37 reacted with MPR.03. The reactivities of 340 *IGHV2/IGHV3* B-CLL cultures (17 patients) for these epitopes were 3, 2, and 1 well, respectively (p<0.0001, p = 0.0007, and p<0.0001; Fisher's exact test vs. the *IGHV*1-69 group). Data are expressed in number of wells positive for each test antigen. NA, not applicable. “-” denotes no binding. ^1^ *IGHV* and *IGKV/IGLV* mutation frequencies (%) were compared with germline according to IMGT. ^2^Two B-CLL mAbs were isolated from separate experiments (Hwang et al., 2012), and the results for binding activity were obtained from the purified IgM paraproteins. ^3^HCDR3 subset numbers were assigned using previously described methods [14].
(TIF)

Figure S2 **Binding characteristics of healthy control B cell cultures.** We stimulated PBMCs from 20 healthy control subjects with EBV using the methods as previously described [28], and the cells were plated at 5,000 cells per well in total of 20 wells per sample. To profile binding characteristics of IgMs, we screened the culture supernatants in ELISA. HIV-1 antigens included aldrithol-2 (AT-2)-inactivated HIV-1 virions ADA (Clade B); HIV-1 group M consensus Env, ConS gp140; and deglycosylated JRFL gp140. HIV-1 Env gp41 linear epitope peptides included HR-1 region peptide, DP107 (NNLLRAIEAQQHLLQLTVWGIKQLQARILAVERYLKDQ); Env clade B HR-2 region peptide, MPER656 (NEQELLELDKWASLWNWFNITNWLW); and Env clade C HR-2 region peptide, MPR.03 (KKKNEQELLELDKWASLWNWFDITNWLWYIRKKK). The reactivities of 400 cultures from 20 non-CLL control subjects for DP107, MPER656, and MPR.03 were 2, 10, and 4 wells, respectively (p<0.0001, p = 0.14, and p<0.0001; Fisher's exact test vs. the *IGHV*1-69 group). Data are expressed in number of wells positive for each test antigen. NA, not applicable.
(TIF)

Table S1 **Immunoglobulin sequence characteristics of B-CLL samples.**
(DOCX)

Table S2 **Summary of B-CLL IgM samples that reacted with HIV-1, HCV, and influenza.**
(DOCX)

Table S3 **Lack of HIV-1 and hepatitis C neutralization by B-CLL IgM paraproteins and the corresponding recombinant IgG_1_ mAbs.**
(DOCX)

Table S4 **Lack of HIV-1 virion capture by B-CLL IgM mAbs.**
(DOCX)

Table S5 **Lack of HIV-1 virion capture by B-CLL recombinant IgG_1_ mAbs.**
(DOCX)

## References

[1] Tomaras GD, Yates NL, Liu P, Qin L, Fouda GG, et al (2008) Initial B-cell responses to transmitted human immunodeficiency virus type 1: virion-binding immunoglobulin M (IgM) and IgG antibodies followed by plasma anti-gp41 antibodies with ineffective control of initial viremia. J Virol 82: 12449–12463. 18842730 10.1128/JVI.01708-08 PMC2593361

[2] Liao HX, Chen X, Munshaw S, Zhang R, Marshall DJ, et al (2011) Initial antibodies binding to HIV-1 gp41 in acutely infected subjects are polyreactive and highly mutated. J Exp Med 208: 2237–2249. 21987658 10.1084/jem.20110363 PMC3201211

[3] Chiorazzi N, Rai KR, Ferrarini M (2005) Chronic lymphocytic leukemia. N Engl J Med 352: 804–815. 15728813 10.1056/NEJMra041720

[4] Fais F, Ghiotto F, Hashimoto S, Sellars B, Valetto A, et al (1998) Chronic lymphocytic leukemia B cells express restricted sets of mutated and unmutated antigen receptors. J Clin Invest 102: 1515–1525. 9788964 10.1172/JCI3009 PMC509001

[5] Ghiotto F, Fais F, Albesiano E, Sison C, Valetto A, et al (2006) Similarities and differences between the light and heavy chain Ig variable region gene repertoires in chronic lymphocytic leukemia. Mol Med 12: 300–308. 17380195 10.2119/2006-00080.Ghiotto PMC1829199

[6] Herve M, Xu K, Ng YS, Wardemann H, Albesiano E, et al (2005) Unmutated and mutated chronic lymphocytic leukemias derive from self-reactive B cell precursors despite expressing different antibody reactivity. J Clin Invest 115: 1636–1643. 15902303 10.1172/JCI24387 PMC1088018

[7] Messmer BT, Albesiano E, Efremov DG, Ghiotto F, Allen SL, et al (2004) Multiple distinct sets of stereotyped antigen receptors indicate a role for antigen in promoting chronic lymphocytic leukemia. J Exp Med 200: 519–525. 15314077 10.1084/jem.20040544 PMC2211936

[8] Murray F, Darzentas N, Hadzidimitriou A, Tobin G, Boudjogra M, et al (2008) Stereotyped patterns of somatic hypermutation in subsets of patients with chronic lymphocytic leukemia: implications for the role of antigen selection in leukemogenesis. Blood 111: 1524–1533. 17959859 10.1182/blood-2007-07-099564

[9] Tobin G, Thunberg U, Karlsson K, Murray F, Laurell A, et al (2004) Subsets with restricted immunoglobulin gene rearrangement features indicate a role for antigen selection in the development of chronic lymphocytic leukemia. Blood 104: 2879–2885. 15217826 10.1182/blood-2004-01-0132

[10] Widhopf GF 2nd, Rassenti LZ, Toy TL, Gribben JG, Wierda WG, et al (2004) Chronic lymphocytic leukemia B cells of more than 1% of patients express virtually identical immunoglobulins. Blood 104: 2499–2504. 15217828 10.1182/blood-2004-03-0818

[11] Chiorazzi N, Ferrarini M (2011) Cellular origin(s) of chronic lymphocytic leukemia: cautionary notes and additional considerations and possibilities. Blood 117: 1781–1791. 21148333 10.1182/blood-2010-07-155663 PMC3056635

[12] Griffin DO, Holodick NE, Rothstein TL (2011) Human B1 cells in umbilical cord and adult peripheral blood express the novel phenotype CD20+ CD27+ CD43+ CD70. J Exp Med 208: 67–80. 21220451 10.1084/jem.20101499 PMC3023138

[13] Seifert M, Sellmann L, Bloehdorn J, Wein F, Stilgenbauer S, et al (2012) Cellular origin and pathophysiology of chronic lymphocytic leukemia. J Exp Med 209: 2183–2198. 23091163 10.1084/jem.20120833 PMC3501361

[14] Darzentas N, Hadzidimitriou A, Murray F, Hatzi K, Josefsson P, et al (2010) A different ontogenesis for chronic lymphocytic leukemia cases carrying stereotyped antigen receptors: molecular and computational evidence. Leukemia 24: 125–132. 19759557 10.1038/leu.2009.186

[15] Baumgarth N (2011) The double life of a B-1 cell: self-reactivity selects for protective effector functions. Nat Rev Immunol 11: 34–46. 21151033 10.1038/nri2901

[16] Baumgarth N, Herman OC, Jager GC, Brown L, Herzenberg LA (1999) Innate and acquired humoral immunities to influenza virus are mediated by distinct arms of the immune system. Proc Natl Acad Sci U S A 96: 2250–2255. 10051627 10.1073/pnas.96.5.2250 PMC26769

[17] Carbonari M, Caprini E, Tedesco T, Mazzetta F, Tocco V, et al (2005) Hepatitis C virus drives the unconstrained monoclonal expansion of VH1-69-expressing memory B cells in type II cryoglobulinemia: a model of infection-driven lymphomagenesis. J Immunol 174: 6532–6539. 15879157 10.4049/jimmunol.174.10.6532

[18] Kipps TJ, Tomhave E, Pratt LF, Duffy S, Chen PP, et al (1989) Developmentally restricted immunoglobulin heavy chain variable region gene expressed at high frequency in chronic lymphocytic leukemia. Proc Natl Acad Sci U S A 86: 5913–5917. 2503826 10.1073/pnas.86.15.5913 PMC297741

[19] Sasso EH, Johnson T, Kipps TJ (1996) Expression of the immunoglobulin VH gene 51p1 is proportional to its germline gene copy number. J Clin Invest 97: 2074–2080. 8621797 10.1172/JCI118644 PMC507282

[20] Abecasis GR, Auton A, Brooks LD, DePristo MA, Durbin RM, et al (2012) An integrated map of genetic variation from 1,092 human genomes. Nature 491: 56–65. 23128226 10.1038/nature11632 PMC3498066

[21] Corti D, Suguitan AL Jr, Pinna D, Silacci C, Fernandez-Rodriguez BM, et al (2010) Heterosubtypic neutralizing antibodies are produced by individuals immunized with a seasonal influenza vaccine. J Clin Invest 120: 1663–1673. 20389023 10.1172/JCI41902 PMC2860935

[22] Throsby M, van den Brink E, Jongeneelen M, Poon LL, Alard P, et al (2008) Heterosubtypic neutralizing monoclonal antibodies cross-protective against H5N1 and H1N1 recovered from human IgM+ memory B cells. PLoS One 3: e3942. 19079604 10.1371/journal.pone.0003942 PMC2596486

[23] Wrammert J, Koutsonanos D, Li GM, Edupuganti S, Sui J, et al (2011) Broadly cross-reactive antibodies dominate the human B cell response against 2009 pandemic H1N1 influenza virus infection. J Exp Med 208: 181–193. 21220454 10.1084/jem.20101352 PMC3023136

[24] Sui J, Hwang WC, Perez S, Wei G, Aird D, et al (2009) Structural and functional bases for broad-spectrum neutralization of avian and human influenza A viruses. Nat Struct Mol Biol 16: 265–273. 19234466 10.1038/nsmb.1566 PMC2692245

[25] Gustchina E, Li M, Louis JM, Anderson DE, Lloyd J, et al (2010) Structural basis of HIV-1 neutralization by affinity matured Fabs directed against the internal trimeric coiled-coil of gp41. PLoS Pathog 6: e1001182. 21085615 10.1371/journal.ppat.1001182 PMC2978731

[26] Luftig MA, Mattu M, Di Giovine P, Geleziunas R, Hrin R, et al (2006) Structural basis for HIV-1 neutralization by a gp41 fusion intermediate-directed antibody. Nat Struct Mol Biol 13: 740–747. 16862157 10.1038/nsmb1127

[27] Sabin C, Corti D, Buzon V, Seaman MS, Lutje Hulsik D, et al (2010) Crystal structure and size-dependent neutralization properties of HK20, a human monoclonal antibody binding to the highly conserved heptad repeat 1 of gp41. PLoS Pathog 6: e1001195. 21124990 10.1371/journal.ppat.1001195 PMC2987821

[28] Hwang KK, Chen X, Kozink DM, Gustilo M, Marshall DJ, et al (2012) Enhanced outgrowth of EBV-transformed chronic lymphocytic leukemia B cells mediated by coculture with macrophage feeder cells. Blood 119: e35–44. 22160618 10.1182/blood-2011-08-371203 PMC3286354

[29] Liao HX, Levesque MC, Nagel A, Dixon A, Zhang R, et al (2009) High-throughput isolation of immunoglobulin genes from single human B cells and expression as monoclonal antibodies. J Virol Methods 158: 171–179. 19428587 10.1016/j.jviromet.2009.02.014 PMC2805188

[30] Rossio JL, Esser MT, Suryanarayana K, Schneider DK, Bess JW Jr, et al (1998) Inactivation of human immunodeficiency virus type 1 infectivity with preservation of conformational and functional integrity of virion surface proteins. J Virol 72: 7992–8001. 9733838 10.1128/jvi.72.10.7992-8001.1998 PMC110135

[31] Liao HX, Sutherland LL, Xia SM, Brock ME, Scearce RM, et al (2006) A group M consensus envelope glycoprotein induces antibodies that neutralize subsets of subtype B and C HIV-1 primary viruses. Virology 353: 268–282. 17039602 10.1016/j.virol.2006.04.043 PMC1762135

[32] Ma BJ, Alam SM, Go EP, Lu X, Desaire H, et al (2011) Envelope deglycosylation enhances antigenicity of HIV-1 gp41 epitopes for both broad neutralizing antibodies and their unmutated ancestor antibodies. PLoS Pathog 7: e1002200. 21909262 10.1371/journal.ppat.1002200 PMC3164629

[33] Chan CH, Hadlock KG, Foung SK, Levy S (2001) V(H)1-69 gene is preferentially used by hepatitis C virus-associated B cell lymphomas and by normal B cells responding to the E2 viral antigen. Blood 97: 1023–1026. 11159532 10.1182/blood.v97.4.1023

[34] Ekiert DC, Bhabha G, Elsliger MA, Friesen RH, Jongeneelen M, et al (2009) Antibody recognition of a highly conserved influenza virus epitope. Science 324: 246–251. 19251591 10.1126/science.1171491 PMC2758658

[35] Kawatsu K, Kumeda Y, Taguchi M, Yamazaki-Matsune W, Kanki M, et al (2008) Development and evaluation of immunochromatographic assay for simple and rapid detection of Campylobacter jejuni and Campylobacter coli in human stool specimens. J Clin Microbiol 46: 1226–1231. 18256225 10.1128/JCM.02170-07 PMC2292965

[36] Schelonka RL, Zemlin M, Kobayashi R, Ippolito GC, Zhuang Y, et al (2008) Preferential use of DH reading frame 2 alters B cell development and antigen-specific antibody production. J Immunol 181: 8409–8415. 19050258 10.4049/jimmunol.181.12.8409 PMC2679994

[37] Catera R, Silverman GJ, Hatzi K, Seiler T, Didier S, et al (2008) Chronic lymphocytic leukemia cells recognize conserved epitopes associated with apoptosis and oxidation. Mol Med 14: 665–674. 19009014 10.2119/2008-00102.Catera PMC2582860

[38] Logvinoff C, Major ME, Oldach D, Heyward S, Talal A, et al (2004) Neutralizing antibody response during acute and chronic hepatitis C virus infection. Proc Natl Acad Sci U S A 101: 10149–10154. 15220475 10.1073/pnas.0403519101 PMC454180

[39] Gorny MK, Wang XH, Williams C, Volsky B, Revesz K, et al (2009) Preferential use of the VH5-51 gene segment by the human immune response to code for antibodies against the V3 domain of HIV-1. Mol Immunol 46: 917–926. 18952295 10.1016/j.molimm.2008.09.005 PMC2693011

[40] Dennison SM, Anasti K, Scearce RM, Sutherland L, Parks R, et al (2011) Nonneutralizing HIV-1 gp41 envelope cluster II human monoclonal antibodies show polyreactivity for binding to phospholipids and protein autoantigens. J Virol 85: 1340–1347. 21106741 10.1128/JVI.01680-10 PMC3020517

[41] Davies J, Riechmann L (1996) Affinity improvement of single antibody VH domains: residues in all three hypervariable regions affect antigen binding. Immunotechnology 2: 169–179. 9373310 10.1016/s1380-2933(96)00045-0

[42] Schroeder HW Jr, Hillson JL, Perlmutter RM (1987) Early restriction of the human antibody repertoire. Science 238: 791–793. 3118465 10.1126/science.3118465

[43] Kipps TJ, Robbins BA, Carson DA (1990) Uniform high frequency expression of autoantibody-associated crossreactive idiotypes in the primary B cell follicles of human fetal spleen. J Exp Med 171: 189–196. 1688607 10.1084/jem.171.1.189 PMC2187670

[44] Kipps TJ, Duffy SF (1991) Relationship of the CD5 B cell to human tonsillar lymphocytes that express autoantibody-associated cross-reactive idiotypes. J Clin Invest 87: 2087–2096. 1710233 10.1172/JCI115239 PMC296965

[45] Kipps TJ, Robbins BA, Kuster P, Carson DA (1988) Autoantibody-associated cross-reactive idiotypes expressed at high frequency in chronic lymphocytic leukemia relative to B-cell lymphomas of follicular center cell origin. Blood 72: 422–428. 3261179

[46] Sasso EH, Willems van Dijk K, Bull AP, Milner EC (1993) A fetally expressed immunoglobulin VH1 gene belongs to a complex set of alleles. J Clin Invest 91: 2358–2367. 8099917 10.1172/JCI116468 PMC443293

[47] Weller S, Braun MC, Tan BK, Rosenwald A, Cordier C, et al (2004) Human blood IgM “memory” B cells are circulating splenic marginal zone B cells harboring a prediversified immunoglobulin repertoire. Blood 104: 3647–3654. 15191950 10.1182/blood-2004-01-0346 PMC2590648

[48] Bomsel M, Heyman M, Hocini H, Lagaye S, Belec L, et al (1998) Intracellular neutralization of HIV transcytosis across tight epithelial barriers by anti-HIV envelope protein dIgA or IgM. Immunity 9: 277–287. 9729048 10.1016/s1074-7613(00)80610-x

[49] Mouquet H, Scheid JF, Zoller MJ, Krogsgaard M, Ott RG, et al (2010) Polyreactivity increases the apparent affinity of anti-HIV antibodies by heteroligation. Nature 467: 591–595. 20882016 10.1038/nature09385 PMC3699875

[50] Lanemo Myhrinder A, Hellqvist E, Sidorova E, Soderberg A, Baxendale H, et al (2008) A new perspective: molecular motifs on oxidized LDL, apoptotic cells, and bacteria are targets for chronic lymphocytic leukemia antibodies. Blood 111: 3838–3848. 18223168 10.1182/blood-2007-11-125450

[51] Morris L, Chen X, Alam M, Tomaras G, Zhang R, et al (2011) Isolation of a human anti-HIV gp41 membrane proximal region neutralizing antibody by antigen-specific single B cell sorting. PLoS One 6: e23532. 21980336 10.1371/journal.pone.0023532 PMC3184076

