# Supplementary figures and images for: IGHV1-69 B Cell Chronic Lymphocytic Leukemia Antibodies Cross-React with HIV-1 and Hepatitis C Virus Antigens as Well as Intestinal Commensal Bacteria
Source: PLoS One. 2014 Mar 10;9(3):e90725. doi: 10.1371/journal.pone.0090725 (PMC3948690; doi:10.1371/journal.pone.0090725)

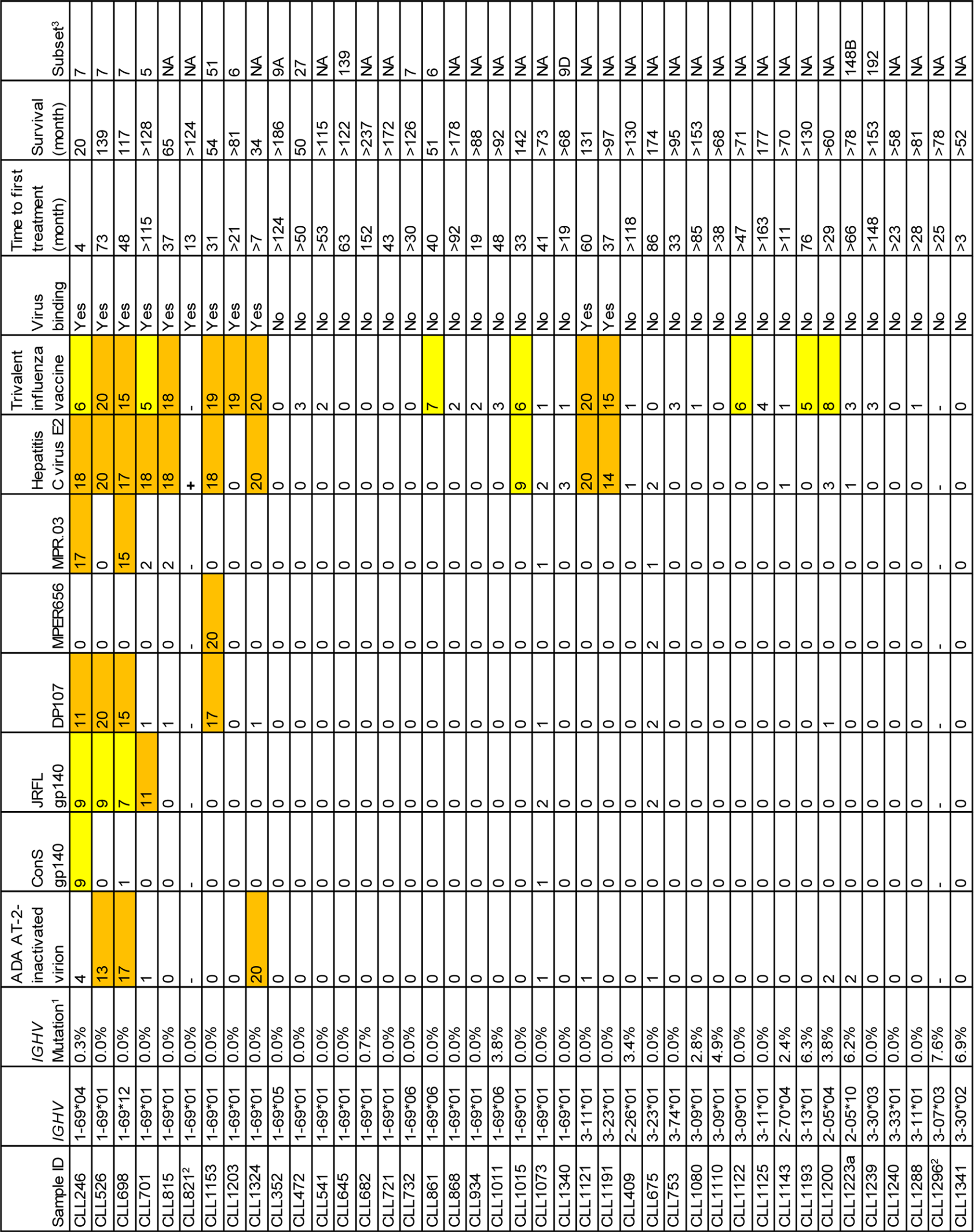

Supplement: Figure S1 — Binding characteristics of B-CLL B cell cultures. To compare binding activities of B-CLL IgMs expressing IGHV1-69 vs. IGHV2/IGHV3 gene families, we stimulated PBMCs from B-CLL patients with EBV using the methods as previously described [28], and the cells were plated at 5,000 cells per well in total of 20 wells per patient sample. To profile binding characteristics of IgMs, we screened the culture supernatants in ELISA. HIV-1 antigens included aldrithol-2 (AT-2)-inactivated HIV-1 virions ADA (Clade B); HIV-1 group M consensus Env, ConS gp140; and deglycosylated JRFL gp140. HIV-1 Env gp41 linear epitope peptides included HR-1 region peptide, DP107 (NNLLRAIEAQQHLLQLTVWGIKQLQARILAVERYLKDQ); Env clade B HR-2 region peptide, MPER656 (NEQELLELDKWASLWNWFNITNWLW); and Env clade C HR-2 region peptide, MPR.03 (KKKNEQELLELDKWASLWNWFDITNWLWYIRKKK). As an initial approach to ensure reactivity of IgMs were of B-CLL origin, rather than IgMs from contaminating B cells, we defined positive samples as they produced 10 or more wells (≥50%) reactive with each test antigen. Of 440 IGHV1-69 B-CLL cultures from 22 patients, 67 wells reacted with DP107, 20 reacted with the MPER656, and 37 reacted with MPR.03. The reactivities of 340 IGHV2/IGHV3 B-CLL cultures (17 patients) for these epitopes were 3, 2, and 1 well, respectively (p<0.0001, p = 0.0007, and p<0.0001; Fisher's exact test vs. the IGHV1-69 group). Data are expressed in number of wells positive for each test antigen. NA, not applicable. “-” denotes no binding. 1 IGHV and IGKV/IGLV mutation frequencies (%) were compared with germline according to IMGT. 2Two B-CLL mAbs were isolated from separate experiments (Hwang et al., 2012), and the results for binding activity were obtained from the purified IgM paraproteins. 3HCDR3 subset numbers were assigned using previously described methods [14]. (TIF) [file pone.0090725.s001.tif]

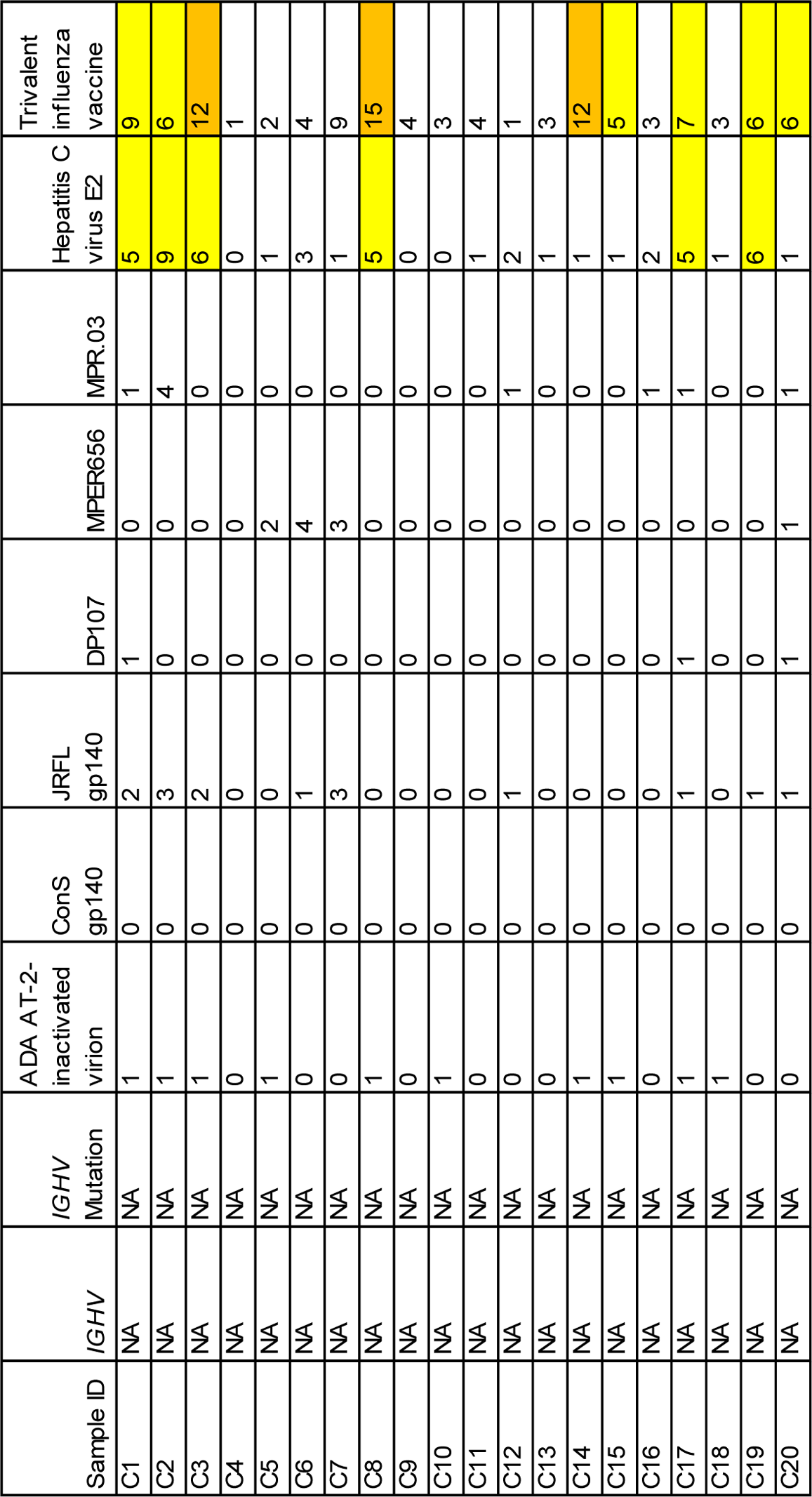

Supplement: Figure S2 — Binding characteristics of healthy control B cell cultures. We stimulated PBMCs from 20 healthy control subjects with EBV using the methods as previously described [28], and the cells were plated at 5,000 cells per well in total of 20 wells per sample. To profile binding characteristics of IgMs, we screened the culture supernatants in ELISA. HIV-1 antigens included aldrithol-2 (AT-2)-inactivated HIV-1 virions ADA (Clade B); HIV-1 group M consensus Env, ConS gp140; and deglycosylated JRFL gp140. HIV-1 Env gp41 linear epitope peptides included HR-1 region peptide, DP107 (NNLLRAIEAQQHLLQLTVWGIKQLQARILAVERYLKDQ); Env clade B HR-2 region peptide, MPER656 (NEQELLELDKWASLWNWFNITNWLW); and Env clade C HR-2 region peptide, MPR.03 (KKKNEQELLELDKWASLWNWFDITNWLWYIRKKK). The reactivities of 400 cultures from 20 non-CLL control subjects for DP107, MPER656, and MPR.03 were 2, 10, and 4 wells, respectively (p<0.0001, p = 0.14, and p<0.0001; Fisher's exact test vs. the IGHV1-69 group). Data are expressed in number of wells positive for each test antigen. NA, not applicable. (TIF) [file pone.0090725.s002.tif]
