# Supplementary material for: IGHV1-69 B Cell Chronic Lymphocytic Leukemia Antibodies Cross-React with HIV-1 and Hepatitis C Virus Antigens as Well as Intestinal Commensal Bacteria
Source: PLoS One. 2014 Mar 10;9(3):e90725. doi: 10.1371/journal.pone.0090725 (PMC3948690; doi:10.1371/journal.pone.0090725)
Supplement: Table S1 — Immunoglobulin sequence characteristics of B-CLL samples. (DOCX) [file pone.0090725.s003.docx]

**Table S1. Immunoglobulin sequence characteristics of B-CLL samples**

^1^*IGHV* and *IGKV/IGLV* mutation frequencies (%) were compared with germline according to IMGT.

^2^HCDR3 and LCDR3 lengths indicate numbers of aa residues.

^3^HCDR3 subset numbers were assigned using previously described methods [14].

^4^The sets of CLL antibody sequences were previously reported [28]. NA, not applicable; nd, not determined.
