# Supplementary material for: IGHV1-69 B Cell Chronic Lymphocytic Leukemia Antibodies Cross-React with HIV-1 and Hepatitis C Virus Antigens as Well as Intestinal Commensal Bacteria
Source: PLoS One. 2014 Mar 10;9(3):e90725. doi: 10.1371/journal.pone.0090725 (PMC3948690; doi:10.1371/journal.pone.0090725)
Supplement: Table S2 — Summary of B-CLL IgM samples that reacted with HIV-1, HCV, and influenza. (DOCX) [file pone.0090725.s004.docx]

**Table S2. Summary of B-CLL IgM samples that reacted with HIV-1, HCV, and influenza**

|  |  | | **HIV-1** | | | | | | **HCV** | **Influenza** |
| --- | --- | --- | --- | --- | --- | --- | --- | --- | --- | --- |
|  |  |  |  |  |  | **gp41 peptides** | | |  |  |
| **Group** | **Samples** | **Total (n)** | **ADA AT-2-inactivated virion** | **ConS gp140** | **Deglycosylated JRFL gp140** | **DP107** | **MPER656** | **MPR.03** | **E2** | **Trivalent influenza vaccine** |
| *IGHV1*-69 | PBMC | 22 | 3 (13.6%) | 0 | 1 (4.5%) | 4 (18.2%) | 1 (4.5%) | 2 (9.1%) | 7 (31.8%) | 6 (27.3%) |
| *IGHV*2/3 | PBMC | 17 | 0 | 0 | 0 | 0 | 0 | 0 | 2 (11.8%) | 2 (11.8%) |
| Controls | PBMC | 20 | 0 | 0 | 0 | 0 | 0 | 0 | 0^1^ | 3 (15.0%) |

^1^p < 0.01 versus the *IGHV*1-69 group (Fisher’s exact test).
