# Supplementary material for: IGHV1-69 B Cell Chronic Lymphocytic Leukemia Antibodies Cross-React with HIV-1 and Hepatitis C Virus Antigens as Well as Intestinal Commensal Bacteria
Source: PLoS One. 2014 Mar 10;9(3):e90725. doi: 10.1371/journal.pone.0090725 (PMC3948690; doi:10.1371/journal.pone.0090725)
Supplement: Table S3 — Lack of HIV-1 and hepatitis C neutralization by B-CLL IgM paraproteins and the corresponding recombinant IgG1 mAbs. (DOCX) [file pone.0090725.s005.docx]

**Table S3. Lack of HIV-1 and hepatitis C neutralization by B-CLL IgM paraproteins and the corresponding recombinant IgG_1_ mAbs**

|  | **HIV-1 pseudoviruses tested (TZM-bl)** | | | | **HCV neutralization** | | |
| --- | --- | --- | --- | --- | --- | --- | --- |
| **mAb ID** | **B.SF162** | **B.BG1168** | **B.MN** | **MuLV** | **H77 (genotype 1a)** | |  |
| CLL246 IgM | - | - | - | - | - |  |  |
| CLL526 IgM | - | - | - | - | - |  |  |
| CLL698 IgM | - | - | - | - | - |  |  |
| CLL821 IgM | - | - | - | - | - |  |  |
| CLL1324 IgM | - | - | - | - | - |  |  |
| CLL1296 IgM | - | - | - | - | - |  |  |
| CLL246 IgG_1_ | - | - | - | - | nd |  |  |
| CLL526 IgG_1_ | - | - | - | - | nd |  |  |
| CLL698 IgG_1_ | - | - | - | - | nd |  |  |
| CLL821 IgG_1_ | - | - | - | - | nd |  |  |
| CLL1324 IgG_1_ | - | - | - | - | nd |  |  |

The B-CLL IgM paraproteins and the recombinant IgG_1_ mAbs were tested at 0.02 – 50 µg/ml in a standard TZM-bl assay. No detectable activity (< 50% neutralization) is indicated with “-“. The CLL1296 IgM was used as a negative control antibody. Nd, not determined.
