# Supplementary material for: IGHV1-69 B Cell Chronic Lymphocytic Leukemia Antibodies Cross-React with HIV-1 and Hepatitis C Virus Antigens as Well as Intestinal Commensal Bacteria
Source: PLoS One. 2014 Mar 10;9(3):e90725. doi: 10.1371/journal.pone.0090725 (PMC3948690; doi:10.1371/journal.pone.0090725)
Supplement: Table S4 — Lack of HIV-1 virion capture by B-CLL IgM mAbs. (DOCX) [file pone.0090725.s006.docx]

**Table S4. Lack of HIV-1 virion capture by B-CLL IgM mAbs**

| **mAb ID** | **B.SF162 (w/o CD4)** | **B.SF162 (w/ CD4)** | **B.BG1168 (w/o CD4)** | **B.BG1168 (w/CD4)** |
| --- | --- | --- | --- | --- |
| CLL246 IgM | 0.34 | 0.36 | 0.17 | 0.32 |
| CLL526 IgM | 0.24 | 0.35 | 0.18 | 0.25 |
| CLL698 IgM | 0.56 | 0.36 | 0.33 | 0.17 |
| CLL821 IgM | 0.55 | 0.46 | 0.21 | 0.21 |
| CLL1324 IgM | 0.55 | 0.63 | 0.19 | 0.19 |
| CLL1296 IgM | 0.61 | 0.65 | 0.12 | 0.29 |
| 7B2 | 5.38 | 122 | 5.43 | 15 |

The B-CLL IgM paraproteins were tested at 5 µg/ml in the presence or absence of soluble CD4. Values are p24 concentration in ng/ml from a standard p24 ELISA. The CLL1296 IgM was used as a negative control antibody while the 7B2 IgG was use as a positive control antibody.
