# Supplementary material for: IGHV1-69 B Cell Chronic Lymphocytic Leukemia Antibodies Cross-React with HIV-1 and Hepatitis C Virus Antigens as Well as Intestinal Commensal Bacteria
Source: PLoS One. 2014 Mar 10;9(3):e90725. doi: 10.1371/journal.pone.0090725 (PMC3948690; doi:10.1371/journal.pone.0090725)
Supplement: Table S5 — Lack of HIV-1 virion capture by B-CLL recombinant IgG1 mAbs. (DOCX) [file pone.0090725.s007.docx]

**Table S5. Lack of HIV-1 virion capture by B-CLL recombinant IgG_1_ mAbs**

| **mAb ID** | **B.SF162 (w/o CD4)** | **B.SF162 (w/ CD4)** | **B.BG1168 (w/o CD4)** | **B.BG1168 (w/CD4)** |
| --- | --- | --- | --- | --- |
| CLL246 IgG_1_ | 1.24 | 0.97 | 0.27 | 0.2 |
| CLL526 IgG_1_ | 1.21 | 1.07 | 0.27 | 0.24 |
| CLL698 IgG_1_ | 1.73 | 1.57 | 0.3 | 0.31 |
| CLL821 IgG_1_ | 1.63 | 1.27 | 0.29 | 0.29 |
| CLL1324 IgG_1_ | 2.16 | 2.07 | 0.29 | 0.33 |
| Ab82 | 1.36 | 1.13 | 0.23 | 0.15 |
| 7B2 | 6.15 | 42 | 1.37 | 3.46 |

The B-CLL recombinant IgG_1_ mAbs were tested at 5 µg/ml in the presence or absence of soluble CD4. Values are p24 concentration in ng/ml from a standard p24 ELISA. The Ab82 IgG was used as a negative control antibody while the 7B2 IgG was use as a positive control antibody.
